# Supplementary material for: Identify devices and events from non-IP heterogeneous IoT network traffic
Source: PeerJ Comput Sci. 2024 Oct 16;10:e2363. doi: 10.7717/peerj-cs.2363 (PMC11623214; doi:10.7717/peerj-cs.2363)
Supplement: Supplemental Information 1 [file peerj-cs-10-2363-s001.zip › README_CN.pdf]

- dataset：这个目录包含实验用到的原始抓包文件和经过处理后，供模型使用的序列样本的缓存文件
    - CICIOT2022_optAslabel_zigbee：从原始数据集中按类别分类的pcap文件
    - CICIOT2022_optAslabel_zwave：从原始数据集中按类别分类的.zlf文件，经过zniffer工具转换后得到的csv文件
    - ZLeaks_devtypeAsLabel_zigbee：从原始数据集中按提供的时间戳切割出来，并按类别分类的pcap文件
    - Other：是使用pcap2json_dpkt_cy.py和cy_cut_dataset.py处理后得到的序列样本的缓存文件
- dataset_maker：对应论文中将样本从Pcap文件处理为序列样本的过程
    - pcap2json_config.py：配置文件，可用于指定目标数据集等参数
    - pcap2json_dpkt_cy.py：将不同的流量文件转换为序列样本
    - cy_cut_dataset.py：用于将完整的序列样本，以不同的随机数切割数据集，并保存到缓存文件
    - cy_analysis_dataset.py：对生成为序列样本情况进行统计
- model：实验使用的模型，和论文中训练模型数据的备份
    - models_compare.py：用不同的序列网络构建模型，并进行训练和测试
    - model_tools.py：一些和模型相关的常用函数
    - models_eval.py：将训练的结果收集起来
- share_lib：依赖的公共函数库
