# Supplementary material for: Identify devices and events from non-IP heterogeneous IoT network traffic
Source: PeerJ Comput Sci. 2024 Oct 16;10:e2363. doi: 10.7717/peerj-cs.2363 (PMC11623214; doi:10.7717/peerj-cs.2363)
Supplement: Supplemental Information 1 [file peerj-cs-10-2363-s001.zip › README_EN.pdf]

- **dataset**: This directory contains the original packet capture files used in the experiments and cached files of sequence samples.
  - **CICIOT2022_optAslabel_zigbee**: Pcap files classified by category from the original dataset.
  - **CICIOT2022_optAslabel_zwave**: Csv files classified by category from the original dataset. These files are obtained by converting zlf files using the zniffer tool.
  - **ZLeaks_devtypeAsLabel_zigbee**: Pcap files segmented by provided timestamps and classified by category from the original dataset.
  - **Other**: Cached files of sequence samples obtained after processing with pcap2json_dpkt_cy.py and cy_cut_dataset.py.
- **dataset_maker**: Corresponds to the process of converting samples from Pcap files to sequence samples as described in the paper.
  - **pcap2json_config.py**: Configuration file used to specify target datasets and other parameters.
  - **pcap2json_dpkt_cy.py**: Converts different traffic files into sequence samples.
  - **cy_cut_dataset.py**: Used to split sequence samples into datasets with different random numbers and save them to cached files.
  - **cy_analysis_dataset.py**: Statistics on the generation of sequence samples.
- **model**: Models used in the experiments, and backups of the data for training models in the paper.
  - **models_compare.py**: Constructs models using different sequence networks and performs training and testing.
  - **model_tools.py**: Some commonly used functions related to models.
  - **models_eval.py**: Collects training results.
- **share_lib:** Shared library of dependencies.
